# Job satisfaction among doctors, a multi-faceted subject studied at a tertiary care hospital in Lahore

**DOI:** 10.12669/pjms.313.7402

**Published:** 2015

**Authors:** Khaula Atif, Habib Ullah Khan, Shahzad Maqbool

**Affiliations:** 1 Khaula Atif, MBBS, MCPS (Fam Med.), DPH, DMA. Department of General Administration, Combined Military Hospital, Peshawar Cantonment, Khyber Pakhtun Khwah, Pakistan; 2 Habib Ullah Khan, MBBS, FCPS (Gen Surg.), FCPS (Neurosurg.). Department of Neuro-Surgery, Combined Military Hospital, Abbottabad Cantonment, Khyber Pakhtun Khwah, Pakistan; 3 Dr. Shahzad Maqbool, MBBS, FCPS (ENT), Department of ENT, Combined Military Hospital, Tarbela Cantonment, Khyber Pakhtun Khwah, Pakistan

**Keywords:** Job Satisfaction, Physician Patient Relation, Delivery of Health Care, Pakistan

## Abstract

**Objective:**

To study the level of job satisfaction among doctors serving in a tertiary care hospital in Lahore and ascertain its co-relation with multiple demographic variables which had a profound impact.

**Methods:**

This cross sectional study with non-probability purposive sampling was conducted at Combined Military Hospital, Lahore, from February 2014 to November 2014. Subjects were doctors serving in that hospital for minimum six months duration. Pre-formed questionnaires were distributed to volunteers (average filling time was 3 ½ to 7 minutes). Multiple demographic features were independent variables. Outcome variable was job satisfaction. Statistical analysis was done via descriptive statistics (SPSS 20), data expressed as mean ± standard deviation (SD).

**Results:**

Out of 263 doctors serving in hospital, 203 (77.91%) volunteered to participate; response rate by depositing the filled forms was 47.78% (97 doctors). Amongst the respondents, 10 (10.3%) doctors had below average job satisfaction, 32(33.0%), 21(21.6%), 21(21.6%) and 13(13.3%) had average, above average, well above average and outstanding job satisfaction respectively. There was significant relation between job satisfaction and age group of the doctors (*p* 0.025), education (*p* 0.015), service years (*p* 0.013) income per month (*p*<0.001). There was no significant impact of gender (*p* 0.540), marital status (*p* 0.087), number of children (*p* 0.153), current employment (*p* 0.71), nature of job (*p* 0.204), working hours (*p* 0.089), additional duties *p* 0.421) and socioeconomic class (*p* 0.104) on outcome variable.

**Conclusion:**

A significant number of doctors was found discontented with their job, which may consequently impact their yield/performance. The job satisfaction can be substantially improved if these contributory factors are aptly addressed at all tiers.

## INTRODUCTION

Medicine has long been considered as one of the most sacred and well reputed professions. In Pakistan too, many children aspire to become doctor since their early childhood.^1^ But gradually a physician’s job has lost its charm as it used to be in past. The prime reason of this attenuated job satisfaction is enormous job stress which a doctor undergoes during performance of his job. Albeit, singular stressors at job contribute immensely in abatement of attraction for this erstwhile most preferred profession.

Though there is a general perception that currently doctors are adequately contented with their jobs, yet formal medical research over the subject is meager all over the globe. The same phenomena holds true for Pakistan where excessive psycho-socio stressors have made doctors more prone to deteriorated job satisfaction. In last two decades, a lot of research has been done to evaluate job satisfaction in medical staff.^3^ Happy doctors, as internal customers, can yield healthy patients.^4^ Job satisfaction amongst doctors is the linchpin to get better delivery of health care.

Many developing countries, including Pakistan, have worsening grades of job satisfaction in their doctors.^1^ Even in US, a well-developed country, less than 50% doctors were satisfied with their job.^5^ In Pakistan, proper studies to ascertain this important facets of this sacred profession are scanty. A person cannot be happy with all aspects of his job at one moment of time.^1^ This study was conducted to evaluate the level of job satisfaction in a tertiary care hospital in Lahore, along with analysis of multiple personal and professional demographic factors which may affect the subject parameter. It was assumed that a significant number of doctors must be unsatisfied with the job and various factors might be impacting profoundly. While making future strategies, such factors can be addressed more religiously to enhance physicians’ job satisfaction and thereby improve the qualitative and quantitative clinical output.

## METHODS

Subjects were the doctors (males and females) serving in a tertiary care hospital in Lahore for at least six months duration. To have a wholesome view, doctors hailing from various departments and age groups were selected. The total strength of doctors serving in the hospital was 263 out of which 203 volunteered to participate. Formal approval was taken from ethical committee of the hospital. Written informed consent was taken and pre-formed questionnaire were distributed among the doctors. developed on the basis of personal and professional demographic features of subjects. The average time to fill the questionnaire was 3 ½ to 7 minutes. Dependent variable was job satisfaction. An arbitrary key was provided to quantify job satisfaction subjectively as below average, average, above average, well above average or outstanding. Same scale was used by administration of that hospital to label performance of its doctors on annual basis. So these terms were familiar and user friendly for the respondents. Independent variable were gender, marital status, age, age group, number of children, education, service years, current employment, nature of job, working hours per week, additional duties, income per month (thousands PKR), socioeconomic class. The statistical analysis was done via descriptive statistics of SPSS 20, data expressed as mean ± standard deviation (SD), cross tabulation was done via chi-square and *p*-value less than 0.05 was considered as significant.

## RESULTS

**Fig.1:**
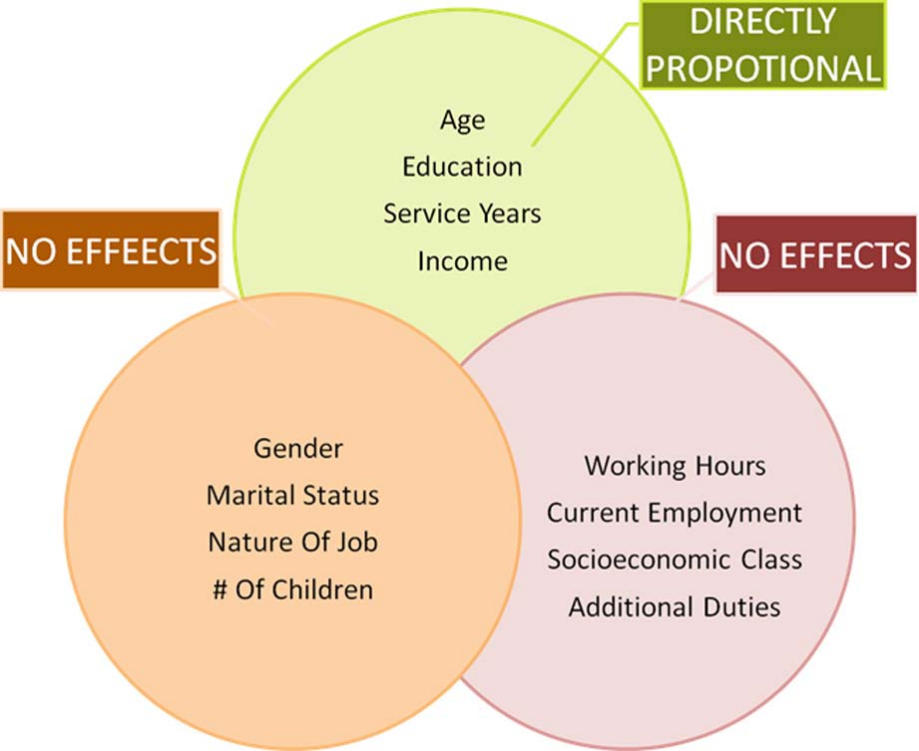
Effects of variables on level of job satisfaction in study participants.

Out of 263 doctors serving in the hospital, 203 (77.91%) volunteered to participate in the study, while only 97 (47.78% of volunteers and 36.88% of doctors serving in the hospital) responded positively by depositing back the filled forms. Demographic details and level of job satisfaction of respondents is tabulated in Table-I and Table-II respectively. There was significant relation between job satisfaction and age group of the doctors (p 0.025), education (p 0.015), service years (p 0.013) income per month (p<0.001); all directly proportional to outcome variable. None of the other variables had a significant impact on dependent variable; gender (p 0.540), marital status (p 0.087), number of children (p 0.153), current employment (p 0.71), nature of job (p 0.204), working hours (p 0.089), additional duties (p 0.421) and socioeconomic class (p 0.104) (Fig.1).

**Table-I:** Demographic Characteristics of Study Participants (Percent).

*1. Marital Status*									
Single	34.0	Once married	66.0	Divorced	Nil	Widowed	Nil	Married more than once	Nil
*2. Age Group*									
22-29 Years	45.4	30-39 years	26.8	40-49 years	16.5	50-59 Years	10.3	60 or more years	1.0
*3. Number Of Children*									
No Child	62.9	1	9.3	2	11.3	3	21.6	4 or more	9.3
*4. Income Per Month In Thousands PKR*									
Honourary	15.5	15-49	32	50-74	21.6	75-99	26.2	100 or more	24.7
*5. Education*									
MBBS	62.9	MCPS and/or MPH	4.1	Msc and/or MPH	5.2	Single FCPS or equivalent	21.6	More than 1 FCPS or equivalent	6.2
*6. Service Years*									
< 4	47.4	5-9	17.5	10-14	7.2	15-19	7.2	20 or more	19.6
*7. Current Employment*									
Army Doctor	52.6	Retired from army and re-employed	5.2	Civilian Doctor	42.2	-	-	-	-
*8. Nature Of Job*									
House Officer	29.9	MO* Administration	3.1	MO* Clinical	17.5	Trainee	23.7	Consultants	25.8
*9. Working Hours Per Week*									
Up to 35	6.2	Up to 95	23.7	Up to 125	13.4	Up to 175 hours	37.1	Up to 245 hours or more	19.6
*10. Additional Duties*									
Nil	7.2	MO**/SMO**	20.6	Resident	46.4	On call from home	16.5	DMO and on call from home	9.3
*11. Socio-Economic Class*									
Low	2.1	Low-Middle	4.1	Middle-Middle	49.5	Upper-Middle	34.0	Upper class	8.2

* Medical Officer.

** Senior Medical Officer.

**Table-II:** Levels of job satisfaction in study participants.

Job Satisfaction	Frequency	Percent
Below Average	10	10.3
Average	32	33.0
Above Average	21	21.6
Well Above Average	21	21.6
Outstanding	13	13.4
Total	97	100.0

## DISCUSSION

This study revealed that a substantial number of doctors were not contented with their job. The subject of job satisfaction amongst doctors has been neglected since long. Doctors face enormous stressors in personal and professional life but high labels associated with this profession like “the Curers” and “the Angels” often deprive them from the intrinsic “human element”. Satisfaction at job is vital to attain superlative quality of work.^6^,^7^ Job satisfaction is simply one’s sensitivity to his work^8^, or how much a person likes or dislikes his job.^9^ A physician’s satisfaction from his job considerably enhances his services and vice versa^10^-^12^ and proportionately affects level of patient’s satisfaction with delivery of health care.^7^,^11^-^13^ Doctor’s behavior with peers and physician patient relation is largely dependent upon level of his job satisfaction.^1^

In this study, 10.3% doctors had below average job satisfaction, 53.6% had average to above average satisfaction, while only 13.3% enjoyed an outstanding satisfaction. Doctors with below average satisfaction cannot always render apt services in routine work as well as in emergency situations. Dissatisfied doctors may be unable to offer a caring and affectionate treatment to their patients; they may also neglect patients due to lack of focus or interest in job. A national study revealed that 26% family physicians in Pakistan were dissatisfied with their profession.^14^ In Karachi, 68% of the doctors were not satisfied with their jobs.^15^ Internationally, in India 26%^10^, and in Australia 14.3%^16^ doctors were unsatisfied from their job. These figures were comparable to our results.

Occupational and non-occupational variables play a vital role to predict job satisfaction and psychological distress in every field.^17^,^18^ A doctor’s job is multifaceted and multi-dimensional. Doctors face unique challenges like difficult and even aggressive patients or attendants, exposure to distressing events, ethical dilemmas and critical decision making which might not be always comprehensible to the patients or their heirs.^15^ Few may develop a guilty feeling; being unable to prevent the death or cure the disease when they had honestly tried to do so. This dissatisfaction is aggravated by lack of respect from the public thus eventually doctors start regretting their choice for this profession.^14^ This research took into consideration various factors which were hypothesized to affect a doctor’s job. It disclosed that plethora of factors were directly proportional to age bracket, education, service years and income per month which had an impact upon their job satisfaction. Gender, marital status, number of children, current employment, nature of job, working hours per week, additional duties and socioeconomic class did not bear significant impact. Other researchers have shown varying results. Some revealed that male doctors were more satisfied with their job^2^,^15^,^19^, others have documented that males were least satisfied^6^, or there was no gender difference.^16^,^20^ Regarding age, some stated youngsters^2^,^20^, or middle aged doctors were least satisfied^6^,^16^, others documented job satisfaction diminishes with age.^19^ Salaries, bonuses and other rewards also affect different doctors in different ways^2^,^6^, affecting almost 70% doctors and their work in some literature.^21^ Large income had a positive impact on satisfaction level.^16^ In Bahawalpur, 56% doctors were not satisfied with their income, 92% were dissatisfied with service structure and career prospects.^1^ In USA, almost 50% doctors felt that they were not adequately paid for their services.^5^ In India, significant number of doctors was unsatisfied with their work environment, average number of working hours and number of night shifts.^22^ In New Zealand, GP’s were not satisfied with the workload and job structure.^23^ Another study documented that physicians were most dissatisfied with the workload and the reforms; they stated that job satisfaction enhances with liberty at work, freedom, salary and benefits like bonuses and salary enhancement.^24^ Literature review did not reveal impact of marital status^19^, level of education^19^, nature of job^16^, and number of working hours on job satisfaction in doctors.^16^

This study has an exception as other similar studies have not yet been undertaken in a tertiary care hospital. It is significant as tertiary care hospital represents physicians from maximum departments of medical field with varying qualifications and service experiences. The questionnaire was relatively simple, user friendly and less time consuming, so chances of neglect in filling the forms can be assumed to be negligible.

## Limitations of Study

First, due to non-probability purposive sampling, the chances of selection bias could not be overruled. Second, generalization of research could not be guaranteed because of low response rate. Respondents could be more study oriented, job conscious or less busy. Third, sample size was small; however, survey response rate was comparable to other similar studies. Fourth, all measures were self reported; limitations in subjective understanding and comprehension could not be denied. Future studies should expand research in primary and secondary care and other tertiary care hospitals of the region, with bigger sample size. For bigger cohort, better questionnaire encompassing more variables can be formulated.

## CONCLUSION

Our initial assumption carried weight as significant number of doctors were found discontented with their jobs. In this regard, myriad factors were found to play a pivotal role in job dissatisfaction of doctors. It is suggested that these overriding factors which impact performance of doctors be addressed at all tires. Consequently, boosted satisfaction at work will warranty better health care delivery and patient welfare, which is the utmost goal of every health care institution.
